# Comparing the therapeutic potentials of *Lactobacillus johnsonii* vs. *Lactobacillus acidophilus* against vulvovaginal candidiasis in female rats: an *in vivo* study

**DOI:** 10.3389/fmicb.2023.1222503

**Published:** 2023-07-17

**Authors:** Dalia Saad ElFeky, Alaa Reda Awad, Asmaa Mohammed Shamseldeen, Hagar Lotfy Mowafy, Sara Adel Hosny

**Affiliations:** ^1^Department of Medical Microbiology and Immunology, Faculty of Medicine, Cairo University, Giza, Egypt; ^2^Department of Physiology, Faculty of Medicine, Cairo University, Cairo, Egypt; ^3^Department of Physiology, Faculty of Medicine, October 6 University, Giza, Egypt; ^4^Histology Department, Faculty of Medicine, Cairo University, Giza, Egypt

**Keywords:** *Lactobacillus acidophilus*, *Lactobacillus johnsonii*, probiotics, rat model, vulvovaginal candidiasis

## Abstract

**Background:**

Vulvovaginal candidiasis (VVC) is a highly prevalent illness affecting women globally. Lactobacilli, which make up the majority of healthy vaginal microbiota (VMB), serve as a powerful barrier against infections. Probiotic therapy has been recommended for the treatment or prevention of VVC.

**Aim of work:**

To compare the *in vivo* therapeutic effects of *Lactobacillus johnsonii* (B-2178) vs. *Lactobacillus acidophilus* (LA-5^®^) on VVC in a rat model, particularly highlighting the immune response of the host vaginal epithelium.

**Methods:**

In total, 30 female Sprague-Dawley rats were divided into 5 groups; Group 1: no intervention, Group 2: ovariectomy group, while animals in Groups 3–5 were subjected to ovariectomy and an intravaginal inoculation of *Candida albicans (C. albicans)* to establish VVC. The animals in Groups 4 and 5 received intravaginal lactobacilli treatment with *L. acidophilus* (LA-5^®^) and *L. johnsonii* (B-2178) strains, respectively, for 7 days. *C. albicans* load was measured in a vaginal lavage 1, 3, and 7 days after the stoppage of the treatment. Histological, morphometric, and immunohistochemical studies of the vaginal tissues were done. IFN-γ, IL-4, and IL-17 were measured in the vaginal tissue.

**Results:**

Both *L. johnsonii* and *L. acidophilus* significantly reduced *C. albicans* vaginal load (250 ± 77.46 and 133.33 ± 40.82 CFU/mL) compared to the count before treatment in both groups (4,850 ± 1419.51 and 4966.67 ± 852.45 CFU/mL) even after 7 days of stoppage of lactobacilli treatment. A statistically significant reduction of the pro-inflammatory cytokines IL-17 and IFN-γ was reported in both treated groups compared to the infected untreated group. *L. johnsonii* has a significant effect on the reduction of hyphae formation of *C. albicans* as well as the nuclear factor kappa B (NF-κB) immunostaining density of vaginal tissue compared to *L. acidophilus.* Moreover, treatment with *L. johnsonii* significantly minimized the epithelium damage triggered by *C. albicans* infection and restored normal vaginal architecture as evidenced by the histologic and morphometric studies when compared to *L. acidophilus.*

**Conclusion:**

Through maintaining an immune tolerant state in the vaginal epithelium and ameliorating the undesirable uncontrolled inflammatory response in the vaginal tissue, *L. johnsonii* (B-2178) has the potential to be utilized alone or in combination with other lactobacilli species in probiotic clinical trials to treat or prevent VVC.

## 1. Introduction

Vaginal microbiota (VMB) represents the host’s first line of defense in maintaining health and preventing infection (Hickey et al., 2012). Vaginal dysbiosis, defined as the alteration of the vaginal microbial community, is usually linked with an increased risk of vaginal infections such as vulvovaginal candidiasis (VVC). VVC is extremely common, affecting almost 75% of women at some point in their lifetime (Denning et al., 2018; Chee et al., 2020). *Candida albicans* (*C. albicans*), a polymorphic opportunistic fungus, is primarily responsible for VVC. It is a typical component of the human microbiota that frequently colonizes the vagina without causing symptoms (Achkar and Fries, 2010). However, symptomatic infection may arise from exaggerated mucosal inflammation that is largely induced by vaginal fungal overgrowth, followed by epithelial invasion and secretion of virulence factors (Willems et al., 2020). Oral or topical azoles are currently used for the treatment of VVC. Nevertheless, this therapy frequently has poor cure rates and is often associated with significant recurrence. Moreover, prolonged use raises the risk of adverse effects and drug resistance (Stabile et al., 2021). Thus, effective alternative therapy for VVC or innovative antifungal medications is urgently needed (Vazquez-Munoz and Dongari-Bagtzoglou, 2021).

Probiotics are live microorganisms that, when given to a host in sufficient amounts, promote health by curing or preventing disease (FAO/WHO, 2006). The most prevalent microorganisms in the VMB of healthy women are lactobacilli. They protect against infections from a variety of pathogens, including *Candida* species (Spaggiari et al., 2022). Potential protective mechanisms include competing for epithelial cell adhesion sites, immune system regulation, and destruction of pathogens by various *Lactobacillus* products such as lactic acid, H_2_O_2_, and bacteriocins (Murina et al., 2014). Since disruption in VMB could cause VVC, several investigations were conducted to determine the therapeutic and preventative efficacy of *Lactobacillus* as a probiotic (XieHY, 2017). Respectively, various species of the *Lactobacillus* complex genus (LCG) have been investigated as probiotic treatments for oral and vulvovaginal *Candida* infections (Vazquez-Munoz and Dongari-Bagtzoglou, 2021). The antifungal activities of *L. acidophilus* against various oral and vaginal *Candida* species have been investigated both *in vitro* and *in vivo* (in animal models) (Gil et al., 2010; Vilela et al., 2015; Salari and Ghasemi Nejad Almani, 2020) where it limited the growth of *C. albicans* through inhibition of biofilm formation and filamentation. Nonetheless, the precise mechanism of their action is still unclear (Salari and Ghasemi Nejad Almani, 2020; Spaggiari et al., 2022). In addition, *L. acidophilus* was evaluated in clinical human trials with variable degrees of effectiveness, to treat or prevent VVC (Murina et al., 2014; Russo et al., 2019; Yefet et al., 2022). Besides its individual role in protection against vaginal dysbiosis, a recent study evaluated the individual and collective effects of different lactobacillus species including *L. acidophilus*. They reported that the probiotic protective function can be produced by interactions between many lactobacillus species and not only by their individual activity (Pacha-Herrera et al., 2022).

Different *Lactobacillus* species and even strains can exhibit unique antifungal activities (Strus et al., 2005; Jang et al., 2019). Hence, research on relatively undiscovered species, like *L. johnsonii* is warranted (Vazquez-Munoz et al., 2022). Although it is closely related to *L. acidophilus*, *L. johnsonii* was initially distinguished from *L. acidophilus* by biochemical and DNA-hybridization studies in 1992 (Fujisawa et al., 1992)*. L. johnsonii* is regarded as a GRAS (generally recognized as safe) microbe along with other lactobacilli (Marcial et al., 2017; Zheng et al., 2020). It is a part of gastrointestinal and vaginal mucosal microbiota; two sites that can be affected by mucosal candidiasis (Fujisawa et al., 1992; Assefa et al., 2015). Several *in vitro* studies showed the antimicrobial activities of *L. johnsonii* and reported that it displays anticandidal properties, through the release of soluble metabolites, that inhibit *C. albicans* planktonic growth as well as biofilm formation (Gil et al., 2010; Zheng et al., 2020; Vazquez-Munoz et al., 2022). In the same context, Abd El-Aala and her team recently investigated the *in vitro* effects of *L. acidophilus* (LA-5) and *L. johnsonii* (B-2178) on *C. albicans* growth, phenotypic and genotypic expression of virulence factors (biofilm and hyphal production). They confirmed that both *L. acidophilus* (LA-5) and *L. johnsonii* (B-2178) were effective in suppressing the growth of *C. albicans*. The cell-free supernatant (CFS) of both species substantially decreased hyphae production, suppressed the expression of virulence-related genes, and limited biofilm formation, but these effects have to be confirmed *in vivo* (Abd El-Aala et al., 2023). A previous study reported the inhibitory effects of *L. johnsonii* on *Gardenella vaginalis* vaginal infection in mice (Joo et al., 2011), but to our knowledge, there are no *in vivo* studies on the inhibitory activity of *L. johnsonii* against VVC in animal models. Therefore, in this study, we aimed to compare the *in vivo* therapeutic effects of *L. johnsonii* (B-2178) vs. *L. acidophilus* (LA-5^®^) on VVC in a rat model, particularly highlighting the immune response of the host vaginal epithelium.

## 2. Materials and methods

The current study was approved by the research ethics committee, October 6 university, approval NO. PRE-Me-2,212,045.

### 2.1. Microorganisms

#### 2.1.1. *Lactobacillus* strains

*Lactobacillus acidophilus* (LA-5^®^) from Chr. Hansen’s collection of dairy cultures (Hrsholm, Denmark) and *L. johnsonii* (B-2178) from the Agricultural Research Service Culture Collection (NRRL - Northern Regional Research Laboratory) (Peoria, Illinois, United States) strains kindly provided by the Department of Dairy Science, Faculty of Agriculture, Cairo University were used in the current study.

#### 2.1.2. *Candida albicans* strain

To study the effect of *L. acidophilus* (LA-5^®^) and *L. johnsonii* (B-2178) strains on VVC, a strain of *C. albicans* isolated from a case of VVC in a previous study (Abd El-Aala et al., 2023) was used to induce the infection in the rat model. The selected strain exhibited the best *in vitro* response to *L. acidophilus* (LA-5) and *L. johnsonii* (B-2178) among the 30 *C. albicans* strains tested by Abd El-Aala et al. (2023).

### 2.2. Animal study

#### 2.2.1. Animals and grouping

**Figure 1:**
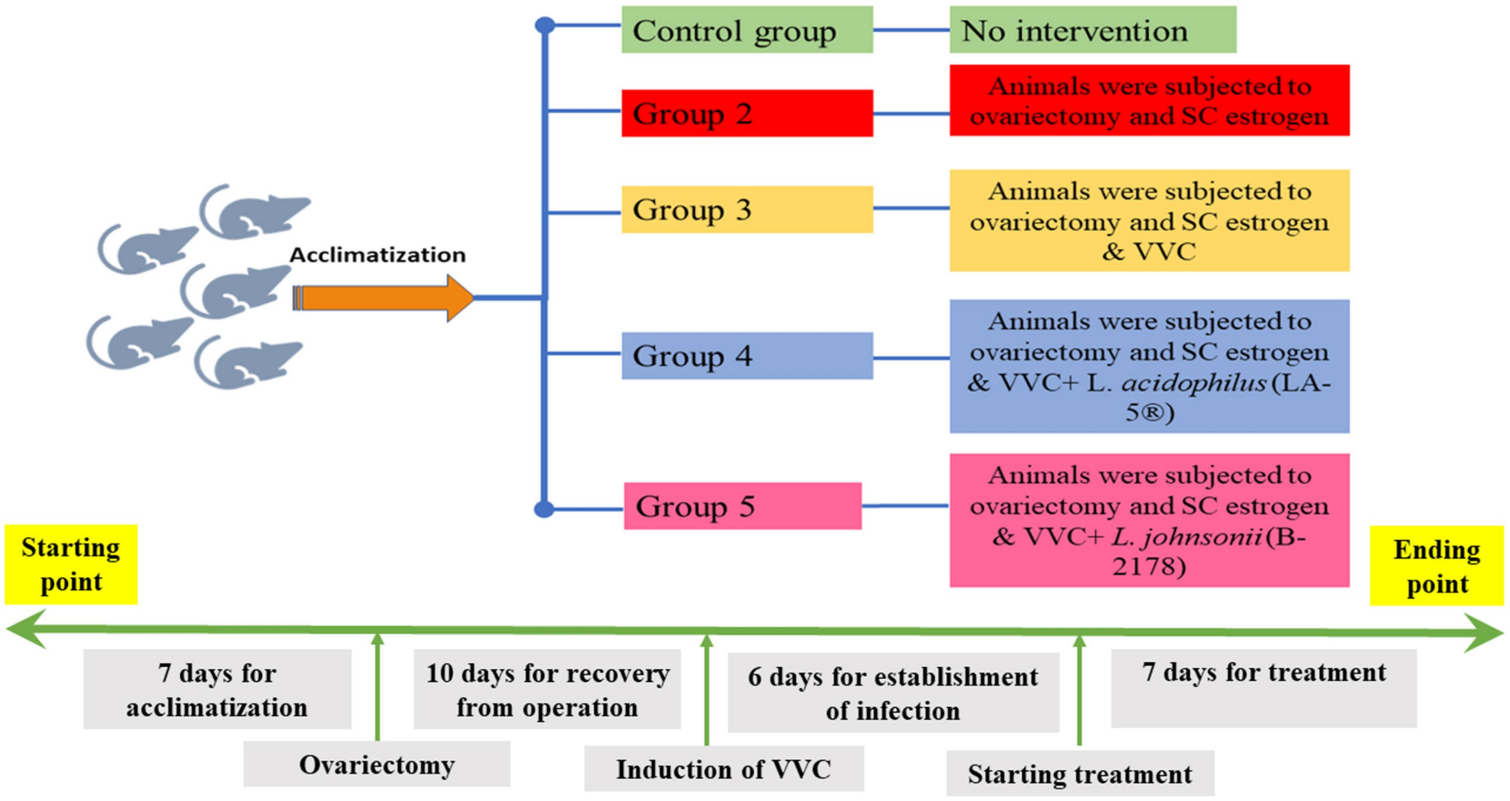
Timeline for the study; a timeline represents all experimental procedures for induction and establishment of vulvovaginal candidiasis (VVC) and scheduled therapeutic intervention using lactobacilli treatment with *L. acidophilus* (LA-5^®^) and *L. johnsonii* (B-2178) strains.

All experimental procedures were carried out according to the International Guidelines of Helsinki. Part of this assurance includes the establishment of an appropriately constituted Institutional Animal Care and Use Committee. All rats were housed in cages with appropriate temperature, humidity, and a 12/12 h light/dark cycle. In total, 30 female Sprague–Dawley rats (weighing 180–200 g) were included in the current study. The animals were divided into 5 groups, each of 6 rats as follows; Group 1: no intervention, Group 2: animals were subjected to ovariectomy and subcutaneous (SC) estrogen administration; and Groups 3–5: animals were subjected to ovariectomy, estrogen administration, and an intravaginal inoculation of *C. albicans* to establish VVC. Animals in Group 3 were the control group for VVC with no treatment, while animals in Groups 4 and 5 received intravaginal lactobacilli treatment with *L. acidophilus* (LA-5^®^) and *L. johnsonii* (B-2178) strains, respectively (Figure 1).

#### 2.2.2. Ovariectomy

After 7 days of adaptation, the animals in Groups 2–5 were subjected to ovariectomy. All surgical procedures were done under complete aseptic precautions. Each female rat was injected with ketamine (50 mg/kg) and xylazine (10 mg/kg) intraperitoneally for induction of anesthesia. After shaving the flank hair off from the last rib down to the pelvis, the skin was disinfected with chlorhexidine solution. Then two separate dorsolateral incisions between the last rib and hips on the right side and the other on the left side were made. After opening the muscle layers, the ovarian pad of fat was pulled out, two knots were done below each ovarian tissue, and then the ovaries were removed (Souza et al., 2019). Finally, the site of the incision was closed using silk material. The post-operative care was done, and animals recovered on the heating pads in their cages and were supplemented with acetaminophen (50 mg/kg) subcutaneously.

#### 2.2.3. Induction of pseudo-estrus state and monitoring of the estrous cycle

Ten days after recovery from the operation, female rats were sustained in a pseudo-estrus state by SC administration of estradiol hexa-hydrobenzoate (0.5 mg/week/rat). The dose was fractioned over three administrations a week, and rats received estradiol until the end of the experiment. Vaginal smear samples were collected daily between 9:00 and 10:00 a.m. at the beginning of the experiment to ensure a normal estrus cycle, after ovariectomy and estrogen administration to confirm the establishment of a pseudo-estrus cycle, and at the end of the experiment to ensure the pseudo-estrus cycle continuity. Smears were prepared by inserting a sterile micropipette into the vaginal opening, flushing deionized water (dH_2_O) 2–3 times in and out, and placing the fluid onto glass slides. The fluid was left in the air to dry and then stained with crystal violet (1%) for 1–2 min. The smears were examined by light microscopy to detect the different cell types, the cornified epithelium, nucleated epithelium, and leukocytes, to determine the estrous stage (Begum et al., 2020). Enucleated cornified cells indicated a pseudo-estrus phase (Mandl, 1951; Carrara et al., 2010).

#### 2.2.4. Establishment of VVC in the animal model

Induction of VVC in the rat model was done as previously described (Li et al., 2019). The selected *C. albicans* strain was subcultured daily on Sabouraud’s dextrose agar for 48 h to get separate colonies. Colonies were harvested and suspended in 0.9% saline solution and adjusted to reach a final concentration of 6 × 10^8^ colony-forming units (CFU)/ml. Infection of the rat model was done by daily intravaginal injection of 500 μL of the prepared *C. albicans* suspension (concentration of 6 × 10^8^ CFU/mL) intravaginally in the animals in Groups 3–5 for 5 days. In parallel, the animals in Groups 1 and 2 received an intravaginal injection of 500 μL sterile saline. On the 6th day of infection, the establishment of VVC was confirmed by microscopic examination and culture of vaginal specimens. For microscopic examination, vaginal swab specimens were obtained, rolled on glass slides, Gram stained, and examined under the light microscope. For the culture procedure, vaginal fluid smear specimens were obtained by vaginal lavage (two consecutive 500 μL volumes of sterile saline). The vaginal lavage specimens were put in sterile tubes and transported immediately to the lab to be subjected to quantitative culture on Sabouraud’s dextrose agar to determine the *C. albicans* load in the vaginal lavage (Li et al., 2019).

#### 2.2.5. Treatment of VVC rat model with lactobacilli

During the treatment phase of the study, both *L. acidophilus* (LA-5^®^) and *L. johnsonii* (B-2178) strains were subcultured daily on MRS agar plates (Himedia, India) anaerobically using Anaerogas Pack (Himedia, India) at 37°C for 48 h to obtain separate colonies. Suspensions were prepared daily by suspending separate colonies of both strains in 0.9% saline solution and suspensions were adjusted to 6 × 10^8^ CFU/mL. After confirming the establishment of VVC by microscopic examination on the 6th day of infection, 500 μL of *L. acidophilus* (LA-5^®^) and *L. johnsonii* (B-2178) suspensions were administered intravaginally to the animals in Groups 4 and 5, respectively, once daily for 7 days. In parallel, the animals in Groups 1–3 received an intravaginal injection of 500 μL of sterile saline (Li et al., 2019).

### 2.3. Assessment of the effect of lactobacilli treatment

The effect of lactobacilli on VVC in rats was assessed by measurement of the *C. albicans* load in the vaginal lavage 1, 3, and 7 days after stoppage of the treatment as described above. At the end of the experiment, all rats were injected intraperitoneally with pentobarbital 150 mg/kg for euthanasia. Whole vagina samples were harvested. The effect of lactobacilli treatment was assessed additionally in the vaginal tissue by histological examination, morphometric and immunohistochemical studies, and measurement of interleukin (IL)-17, IL-4, and interferon (IFN)-γ using enzyme-linked immunosorbent assay (ELISA).

#### 2.3.1. Estimation of IFN-gamma (IFNγ), IL-4, and IL-17 in vaginal tissues

After homogenization of vaginal tissue samples, the levels of IFNγ, IL-4, and IL-17 were estimated in mg tissue protein using Enzyme-Linked Immuno-Sorbent Assay (ELISA) kits. According to the manufacturer’s instructions, the levels of IFNγ were determined using Rat IFNγ ELISA Kit (Catalog # BMS621, Thermofisher, United States), the levels of IL-4 were determined using Rat IL-4 ELISA Kit (ab100771, Abcam, Cambridge, United Kingdom), and the levels of IL-17 were estimated using Rat IL-17 ELISA Kit (MyBioSource, MBS2022678). The levels were estimated in mg tissue protein.

#### 2.3.2. Histological studies

The entire vaginal tube from the experimental groups was dissected, fixed in 10% formal saline, and processed to create paraffin sections of 5–7 m thickness. These sections were then stained with hematoxylin and eosin (H&E), Masson’s trichrome, and Orcin stains.

#### 2.3.3. Immunohistochemical studies

Immunohistochemical staining of vaginal tissue sections was done using the avidin-biotin peroxidase complex technique by anti-NF-κB (aab16502; a rabbit monoclonal antibody, Abcam plc, England). For antigen retrieval, vaginal sections were boiled in citrate buffer pH 6 for 10 min, followed by overnight incubation with the primary antibody. Mayer’s hematoxylin was employed as a counterstain and diaminobenzidine (DAB) as a chromogen. To provide negative control serial sections for immunohistochemical staining specificity, the main antibody was changed to phosphate buffer saline. Megakaryocyte cytoplasmic staining in human thrombocytosis served as a positive tissue control for NF-κB immunostaining.

### 2.4. Statistical analysis

The statistical software for the social sciences (SPSS) version 28 (IBM Corp., Armonk, NY, United States) was used to code and input the data. Data were presented through mean and standard deviation. ANOVA with a *post hoc* test was utilized for group comparisons (Chan, 2003). For the comparison of serial measurements within each group repeated measures ANOVA was used (Chan, 2004). Statistics were considered significant for *p*-values under 0.05.

## 3. Results

### 3.1. Successful induction of a pseudo-estrus state and VVC in the rat model

**Figure 2:**
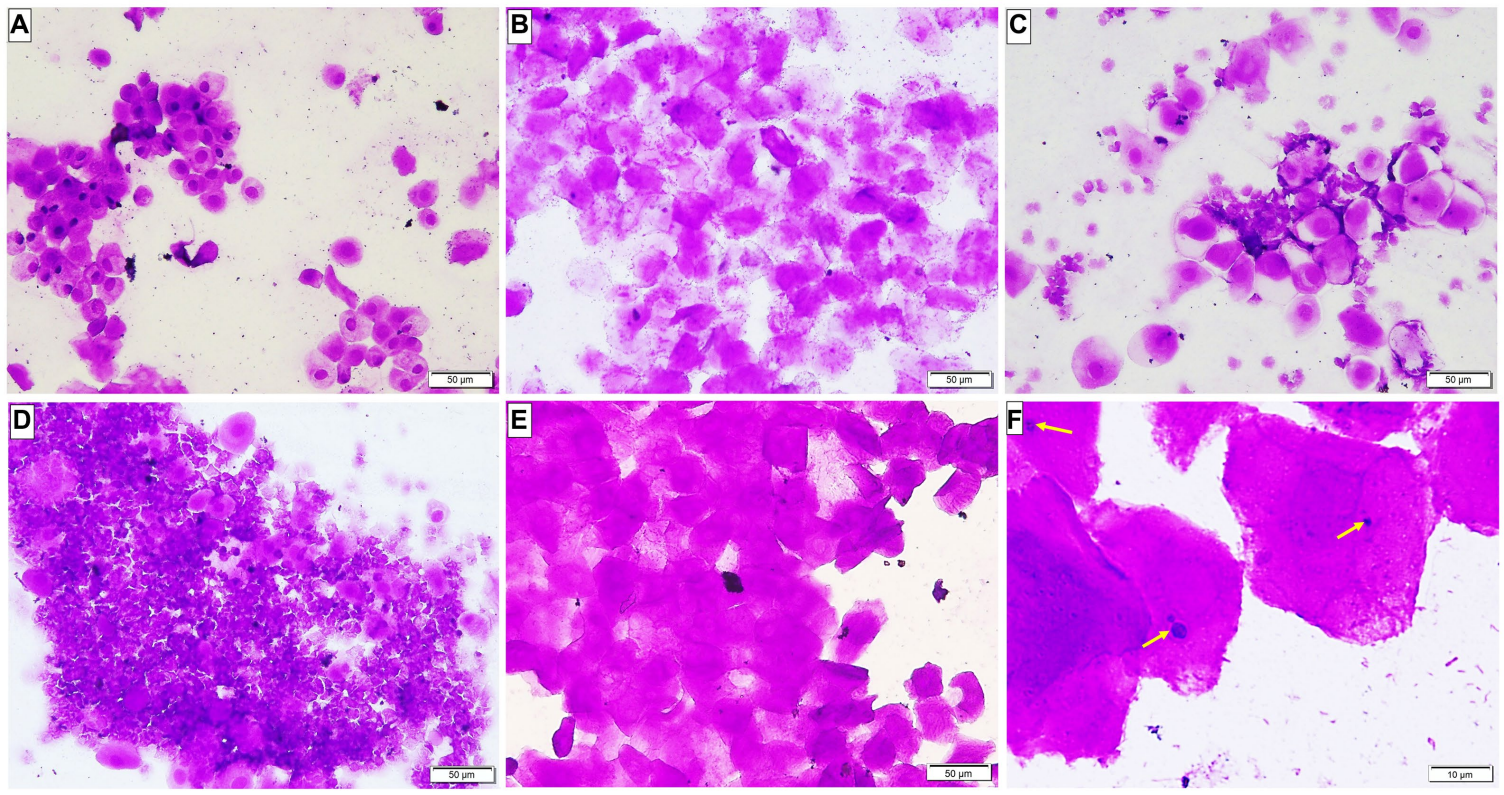
A Photomicrograph of vaginal smears stained with crystal violet-stained (**A–E** ×200, **F** ×400). **(A–D)** Group 1: displaying normal estrous cycles of 4 subsequent phases: **(A)** proestrus phase having nucleated epithelial cells; **(B)** estrus phase having cornified epithelial cells; **(C)** metestrus phase having lymphocytes, epithelial and non-nucleated cells; and **(D)** diestrus phase having a large number of lymphocytes. **(E)** Groups after ovariectomy and estrogen administration: pseudo-estrus phase can be demonstrated. **(F)** Group 3: showing *Candida albicans* in yeast form (yellow arrows).

Microscopic examination of the vaginal smear in all groups at the beginning of the experiment revealed a normal estrus cycle formed of four subsequent phases but after ovariectomy and estrogen administration, a pseudo-estrus phase was established which continued until the end of the experiment. *Candida* infection was confirmed by demonstrating the pathogen in the vaginal smear (Figure 2).

### 3.2. Inhibitory effect of *Lactobacillus acidophilus* (LA-5^®^) and *Lactobacillus johnsonii* (B-2178) on *Candida albicans* in VVC rats

**Figure 3:**
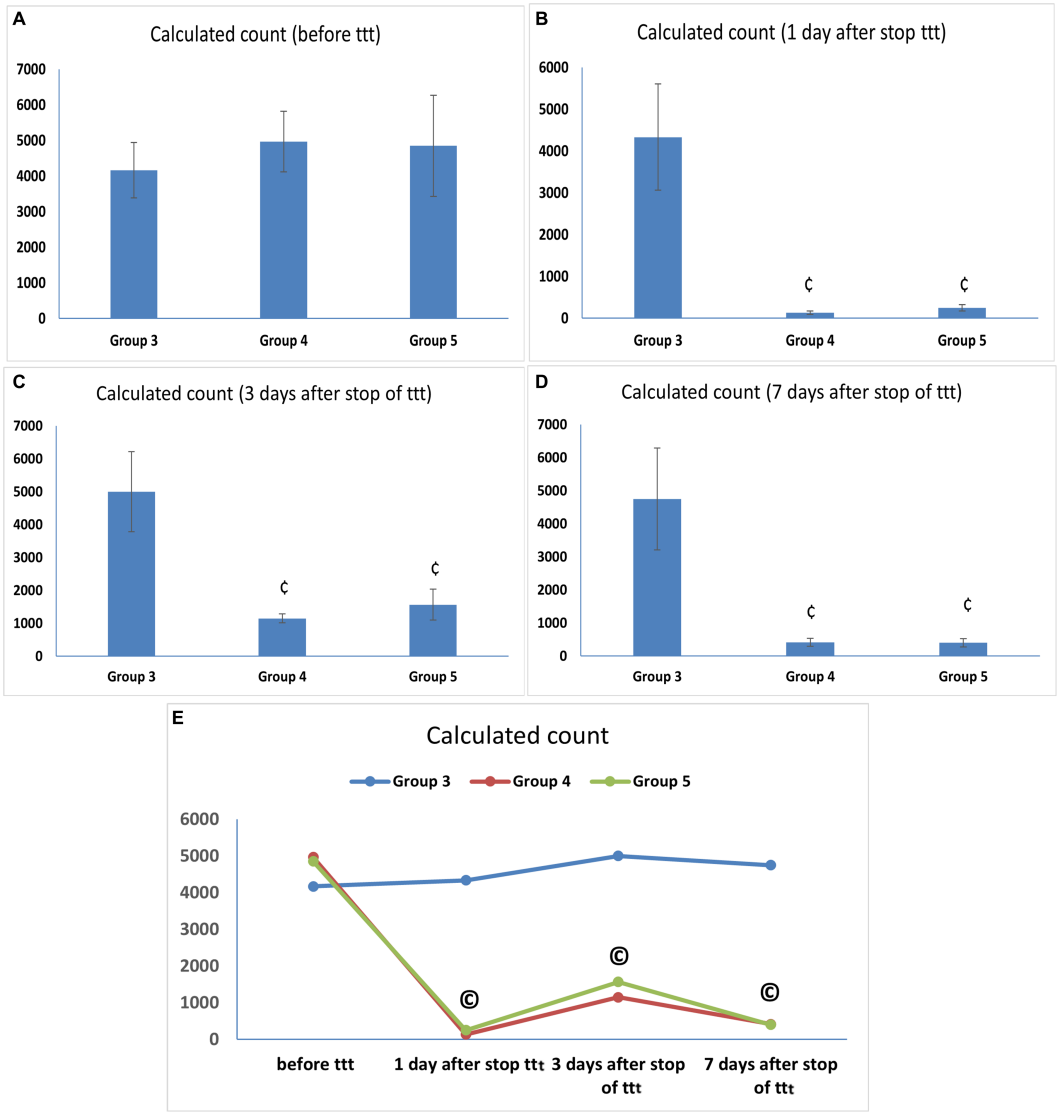
**(A–D)** *Candida albicans* load (CFU/mL) in vaginal lavage specimens. Values are presented as mean ± SD, ¢: statistically significant compared to the corresponding value in Group 3 (*p* < 0.05), **(E)** *Candida albicans* load (CFU/mL) in vaginal lavage specimens in each group over time, data presented as mean values. ^©^: statistically significant compared to the corresponding value before treatment (*p* < 0.05).

At the end of the infection phase and before starting the treatment phase, all vaginal fluid samples from the infected animals were positive for *C. albicans* with a mean count of (4166.67 ± 776.32 CFU/mL) in Group 3 and comparable counts in Groups 4 and 5 (4966.67 ± 852.45 and 4,850 ± 1419.51 CFU/mL) with no statistically significant difference between them. Our results demonstrated comparable potent anticandidal effects for vaginal treatment with either *L. acidophilus* or *L. johnsonii* displayed by the marked drop in *C. albicans* count 1 day after stopping treatment with both species (133.33 ± 40.82 and 250 ± 77.46 CFU/mL, respectively), with a statistically significant difference compared to the corresponding value in Group 3 (*p* < 0.05). This significant anti-*C. albicans* effect was maintained even after 7 days of stoppage of treatment (Supplementary Table S1 and Figures 3A–D). Regarding the assessment of anti-*Candida* activity of lactobacilli over different time points after treatment, there was a statistically significant drop in *C. albicans* counts in Groups 4 and 5 after treatment compared with the corresponding value in the same group before treatment (*p* < 0.05), however, no statistically significant count difference was found between the two lactobacilli treated groups (Groups 4 and 5) over the different time points after stoppage of the lactobacilli treatment (Supplementary Table S1 and Figure 3E).

### 3.3. Effect of *Lactobacillus acidophilus* (LA-5^®^) and *Lactobacillus johnsonii* (B-2178) on cytokine level in vaginal tissue specimens

**Figure 4:**
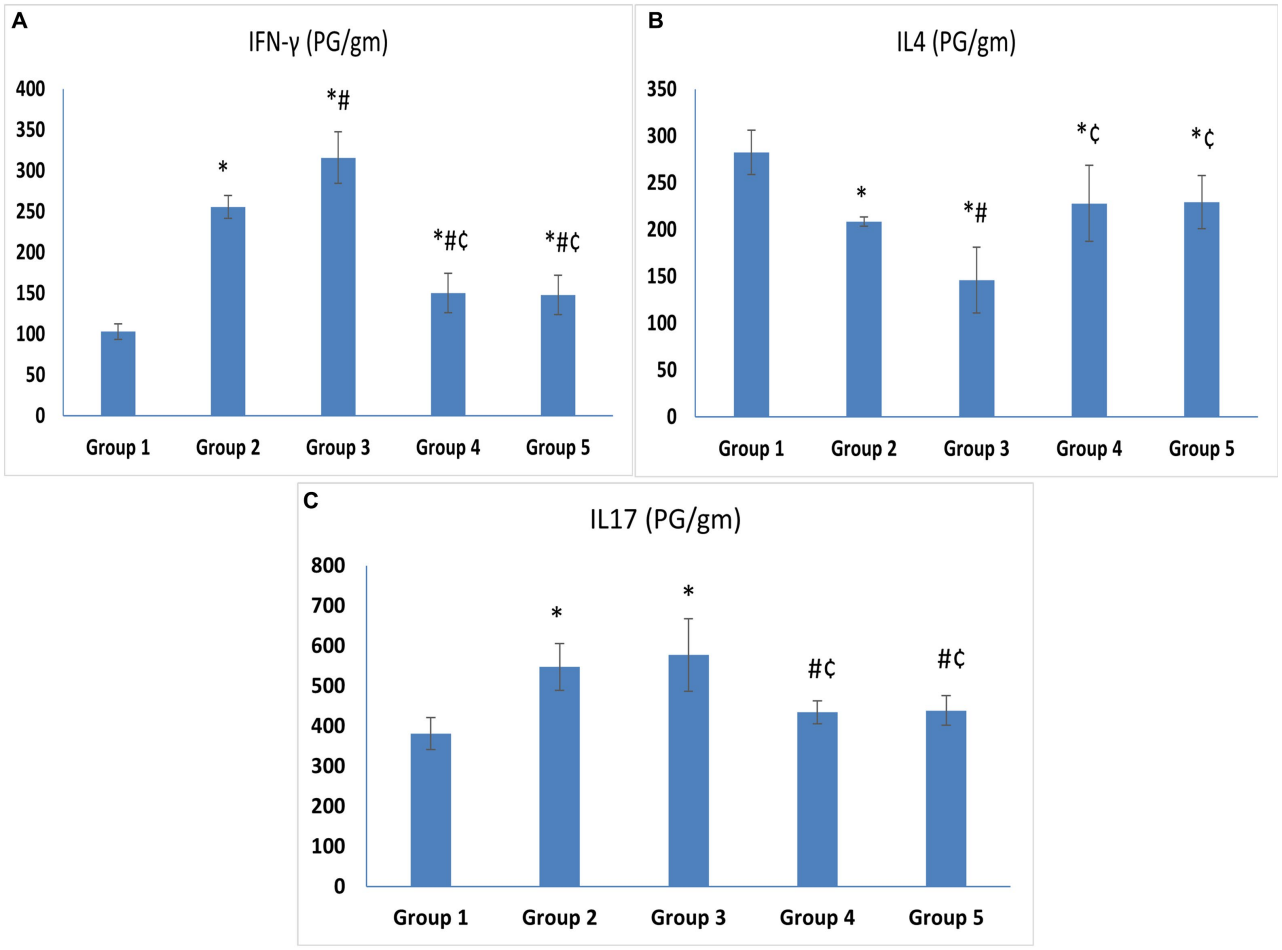
**(A)** IFN-γ. **(B)** IL-4. **(C)** IL-17. Cytokine levels in vaginal tissue specimens. Values are presented as mean ± SD, *: statistically significant compared to the corresponding value in Group 1 (*p* < 0.05), #: statistically significant compared to the corresponding value in Group 2 (*p* < 0.05), ¢: statistically significant compared to the corresponding value in Group 3 (*p* < 0.05).

The levels of IFN-γ, the hallmark of T helper-1 (Th1), and IL-17, the hallmark of Th17, were significantly higher in the untreated VVC group (Group 3) (315.83 ± 31.64 and 577.27 ± 90.46) compared to the control group (Group 1) (102.92 ± 9.34 and 381.55 ± 39.62) (*p* < 0.05). The level of IFN-γ in Group 2 was higher than in Group 1 with a statistically significant difference. On the other hand, the levels of IL-4, the hallmark of Th2, were significantly lower in Group 3 (145.88 ± 35.17) compared to the control group (Group 1) (282.43 ± 23.93) (*p* < 0.05) (Figures 4A–C).

Regarding treatment groups, there was a statistically significant drop in the levels of IFN-γ (150.38 ± 24.07 and 147.65 ± 24.13) and IL-17 (434.57 ± 28.29 and 439 ± 37.37), in the vaginal tissue of the animals treated with *L. acidophilus* and *L. johnsonii,* respectively, compared to Group 3 (*p* < 0.05). Meanwhile, IL-4 was higher in both treated groups compared to Group 3. No statistically significant difference was observed between the *L. acidophilus* and *L. johnsonii* treatment groups regarding levels of IFN-γ, IL-17, or IL-4 (*p* = 1) (Figures 4A–C).

### 3.4. Histological results

**Figure 5:**
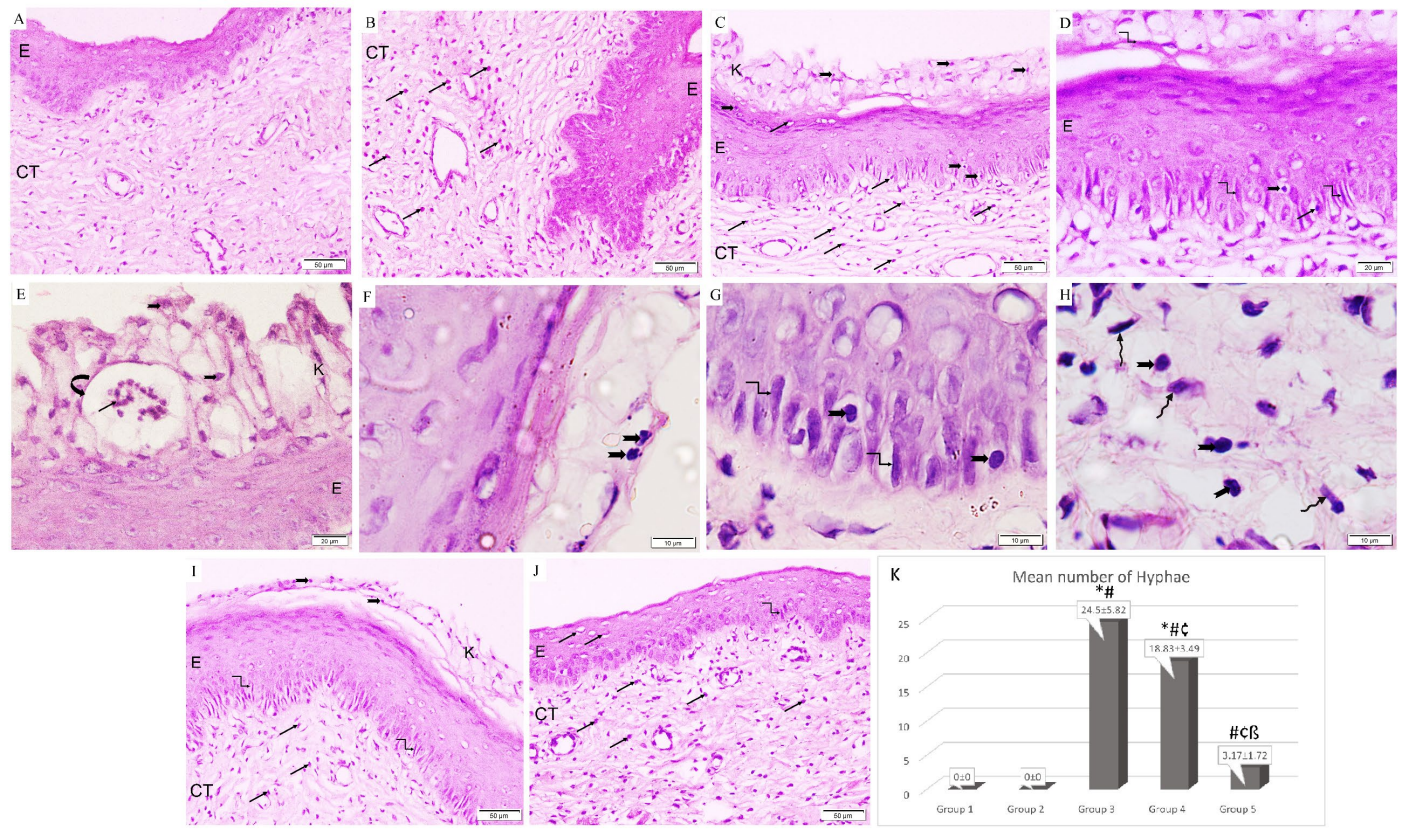
A photomicrograph of rat vaginal sections stained with H&E (**A,B,C,I,J** ×200, **D,E** ×400, and **F–H** ×1,000). **(A)** Group 1: exhibits normal histological architecture of the vagina formed of non-keratinized stratified squamous epithelium (E) lining the lumen with underlying connective tissue stroma (CT). **(B)** Group 2: The lining epithelium (E) is thickened associated with an increased amount of eosinophilic infiltration (arrows) in the underlying connective tissue stroma (CT). **(C–E)** Group 3: keratin layer is dissoluted with the presence of necrotic tissue debris (k) penetrated with *Candida* yeast (notched arrows) and hyphae (kinked arrows). The formation of microabscesses (curved arrow) in the cornified epithelium containing neutrophils (black arrows). Infiltration of *Candida* yeast (notched arrows), hyphae (kinked arrows), and neutrophils (arrows) in the epithelium (E) and underlying connective tissue (CT). **(F–H)** Group 3: higher magnification showing infiltration of *Candida* yeast (notched arrows) and hyphae (kinked arrows) in the cornified epithelium, epithelium, and underlying connective tissue among fibroblasts (wavy arrows). **(I)** Group 4: Necrotic debris is demonstrated in the dissoluted keratin layer (k) with the presence of *Candida* yeast (notched arrows) and the epithelium (E) exhibits hyphae (kinked arrows). Neutrophilic infiltration (arrows) is minimal in the connective tissue (CT). **(J)** Group 5: Epithelium (E) infiltrated with minimal neutrophils (arrows) and hyphae (kinked arrows). Neutrophilic infiltration (arrows) is detected in the underlying connective tissue (CT). **(K)** Histogram showing the mean number of hyphae in the epithelium of studied groups *: statistically significant compared to the corresponding value in Group 1 (*p* < 0.05), #: statistically significant compared to the corresponding value in Group 2 (*p* < 0.05), ¢: statistically significant compared to the corresponding value in Group 3 (*p* < 0.05), ß: statistically significant compared to the corresponding value in Group 4 (*p* < 0.05).

The control group illustrated a normal histological architecture of the vagina (Figure 5A). Following ovariectomy and estrogen treatment, the epithelial lining in the rats in Group 2 was thickened together with an increase in eosinophilic infiltrations in the connective tissue stroma (Figure 5B). However, the rats in Group 3 showed keratin layer disintegration along with necrotic tissue debris that had been infiltrated by *Candida* yeast and hyphae. It is important to note the development of micro-abscess in cornified epithelium along with pronounced infiltration of *Candida* yeast, hyphae, and neutrophils in the epithelium and underlying connective tissue (Figures 5C–H). Similar to Group 3, the *L. acidophilus* (LA-5^®^) treatment group revealed necrotic debris in the disintegrated keratin layer together with *Candida* yeast and hyphae, but with only moderate neutrophil infiltration in the connective tissue (Figure 5I). On the other hand, treatment with *L. johnsonii* showed a substantial reduction in vaginal epithelial mucosa injury and inflammation which nearly returned the vaginal architecture to normal apart from a minor neutrophil infiltration (Figure 5J).

Regarding the effect on hyphae formation, it was observed that Group 3 had the highest mean percentage of hyphae production. Additionally, it was significantly higher in Groups 3 and 4 when compared to the control group, and significantly lower in both treatment groups when compared to Group 3. Moreover, it was significantly reduced in Group 5 in comparison with Group 4 (Figure 5K).

**Figure 6:**
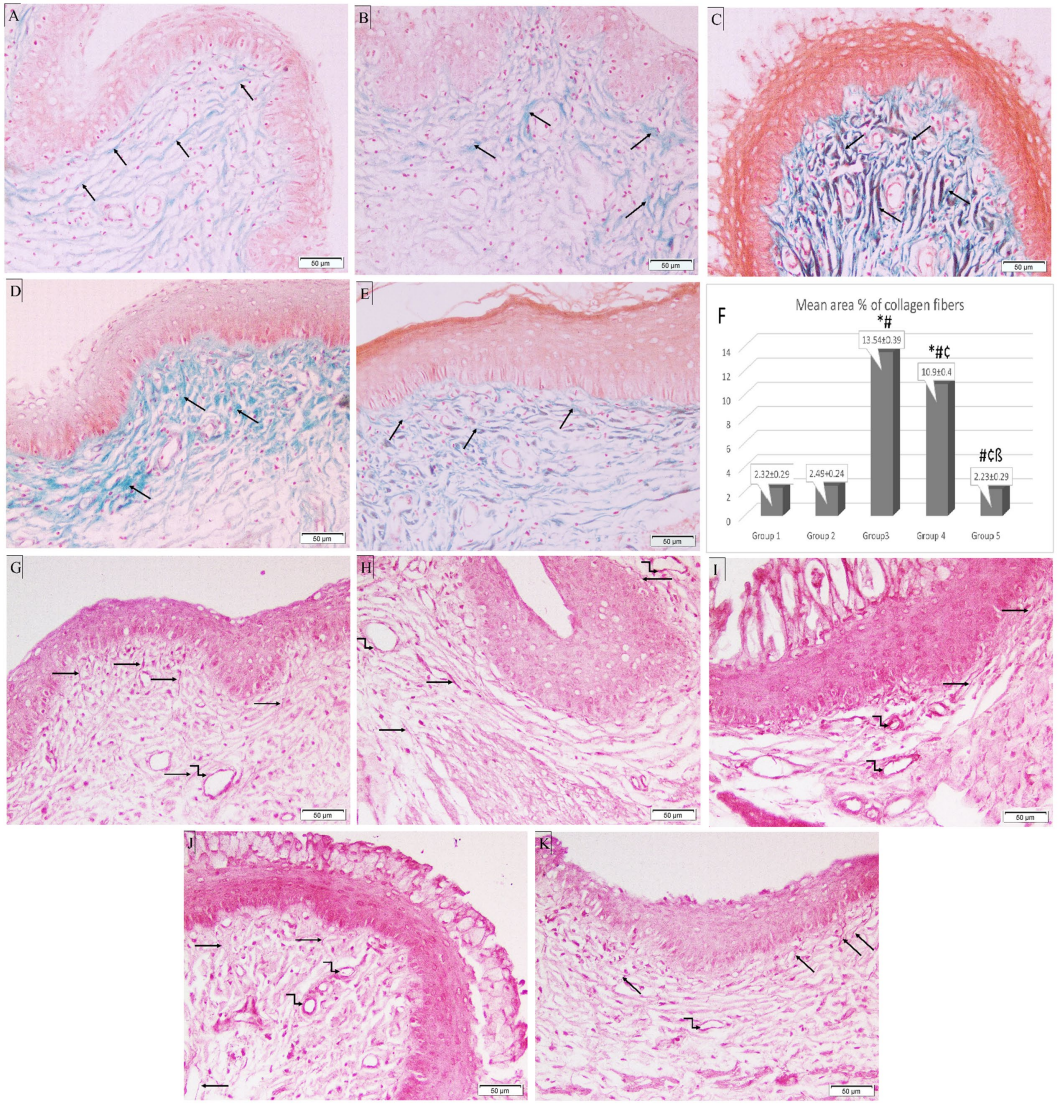
Photomicrographs of special-stained rat vaginal sections (×200). Masson’s trichrome stain: **(A)** Group 1: reveals normal distribution of fine collagen fibers (black arrows) in CT stroma underlying the epithelium. **(B)** Group 2: demonstrates a minimal increase of collagenous fibers (black arrows) in CT stroma underlying the epithelium. **(C)** Group 3: exhibits an abundant number of thick collagen fibers (black arrows) in CT stroma underlying the epithelium. **(D)** Group 4: shows an increased number of collagenous fibers (black arrows) in CT stroma. **(E)** Group 5: exhibits a minimal number of collagen fibers (black arrows) in CT stroma. **(F)** Histogram showing mean area percentage of collagen fibers stained with trichrome stain. *: statistically significant compared to the corresponding value in Group 1 (*p* < 0.05), #: statistically significant compared to the corresponding value in Group 2 (*p* < 0.05), ¢: statistically significant compared to the corresponding value in Group 3 (*p* < 0.05), ß: statistically significant compared to the corresponding value in Group 4 (*p* < 0.05). Orcein stain: **(G)** Group 1: shows long branching brick red elastic fibers (black arrows) in CT stroma with fine elastic fibers in the wall of blood vessels (kinked arrow). **(H)** Group 2: reveals thick elastic fibers (black arrows) in CT stroma and the wall of blood vessels (kinked arrows). **(I)** Group 3: shows few fine elastic fibers (black arrows) in CT stroma with thick elastic fibers around dilated blood vessels (kinked arrows). **(J)** Group 4: exhibits short fine elastic fibers (black arrows) in CT stroma with thick brick red elastic fibers in the wall of blood vessels (kinked arrows). **(K)** Group 5: demonstrates long branching elastic fibers (black arrows) in CT stroma with fine elastic fibers in the blood vessel wall (kinked arrow).

In the control group, Masson’s trichrome staining showed that the tiny collagen fibers beneath the epithelium in the CT stroma were distributed normally. Following ovariectomy, estrogen treatment barely enhanced collagen fibers. The mean area percentage of collagen fibers was significantly increased in Groups 3 and 4 (13.54 ± 0.39 and 10.9 ± 0.4 respectively) compared to the control group (Group 1) (2.32 ± 0.29). On the other hand, it was significantly lower in both treatment groups (Groups 4 and 5) (10.9 ± 0.4 and 2.23 ± 0.29, respectively) compared to Group 3 (13.54 ± 0.39). Additionally, Group 5 showed a significant reduction in the mean area percentage of collagen fibers compared to Group 4 (*p* < 0.05) (Figures 6A–F).

In the CT stroma of the control group, orcein staining revealed long branching brick-red elastic fibers, which were thickened in Group 2. In Group 3, *Candida* infection destroyed the elastic fibers in the CT, leaving only a few thin fibers and thick elastic fibers surrounding dilated blood vessels. Treatment with *L. acidophilus* (LA-5^®^) did not restore elastic fibers, however, *L. johnsonii* (B-2178) treatment demonstrated long branching elastic fibers (Figures 6G–K).

### 3.5. Immunohistochemistry of vaginal sections

**Figure 7:**
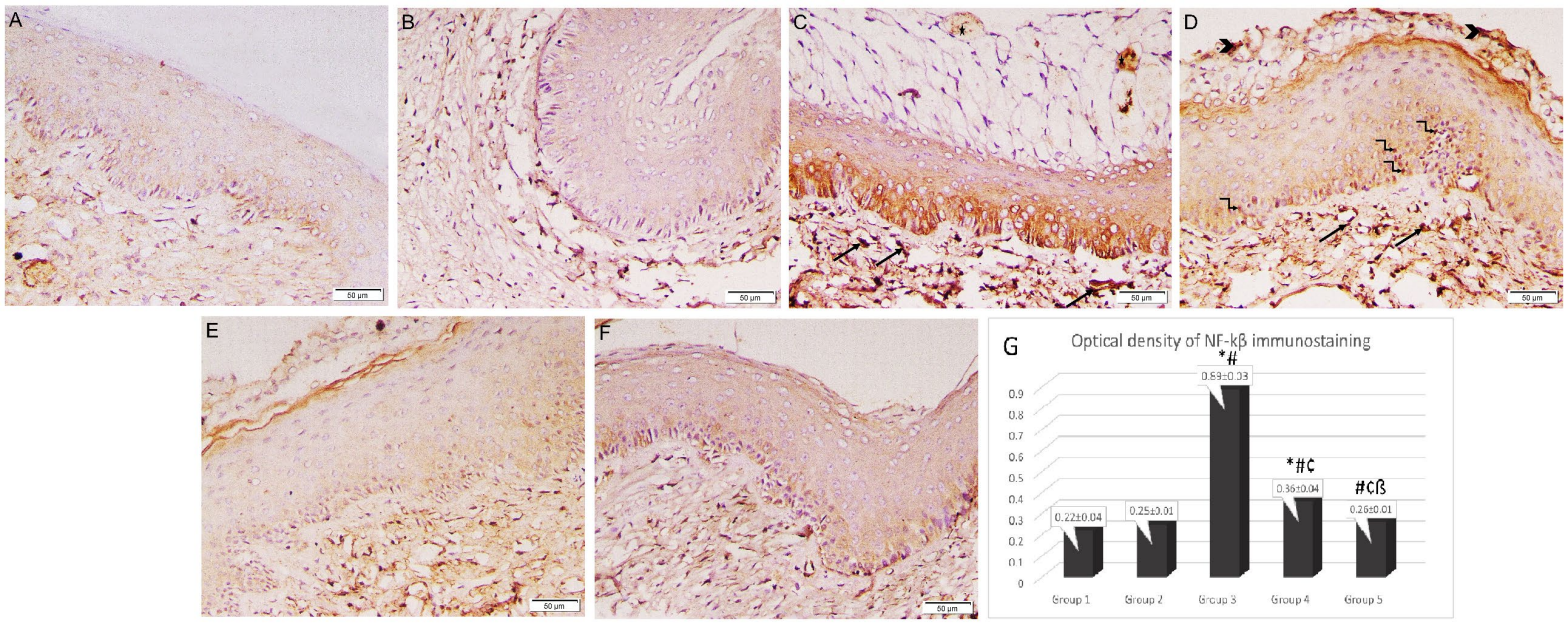
Photomicrographs of rat vaginal sections stained immunohistochemically with NF-κB (×200). **(A)** Group 1: weak positive cytoplasmic NF-κB immunostaining in epithelial and stromal cells. **(B)** Group 2: positive cytoplasmic NF-κB immunostaining in epithelial and stromal cells. **(C,D)** Group 3: intense widespread positive cytoplasmic NF-κB immunostaining in epithelial, stromal cells associated with positive nuclear reaction in some epithelial cells (kinked arrows), stromal cells (arrows) and necrotic tissue debris (arrowheads). The positive reaction of the microabcess in the cornified epithelium (asterisk). **(E,F)** Groups 4 and 5: mild positive cytoplasmic NF-κB immunostaining in epithelial and stromal cells. **(G)** Histogram showing the optical density of NF-κB immunostaining in the studied groups *: statistically significant compared to the corresponding value in Group 1 (*p* < 0.05), #: statistically significant compared to the corresponding value in Group 2 (*p* < 0.05), ¢: statistically significant compared to the corresponding value in Group 3 (*p* < 0.05), ß: statistically significant compared to the corresponding value in Group 4 (*p* < 0.05).

In immunohistochemical examination of the vaginal sections, epithelial and stromal cells in Groups 1 and 2 exhibited weakly positive cytoplasmic immunostaining for NF-κB. Intense, pervasive positive cytoplasmic and nuclear immunostaining was observed in the Group 3 sections in the epithelial, stromal, and necrotic tissue debris, along with a positive reaction of micro-abscess in the cornified epithelium. Groups 3 and 4 had a significantly higher optical density of NF-κB immunostaining (0.89 ± 0.03 and 0.36 ± 0.04 respectively) when compared to the control group (Group 1) (0.22 ± 0.04). However, the intensity of NF-κB immunostaining was significantly reduced in the lactobacilli-treated groups (Groups 4 and 5) (0.36 ± 0.04 and 0.26 ± 0.01 respectively) when compared to Group 3 (0.89 ± 0.03) (*p* < 0.05). Additionally, Group 5 revealed a marked reduction in NF-κB immunostaining compared to Group 4 (*p* < 0.05) (Figure 7).

## 4. Discussion

Mucosal surfaces are the main interface between the host and its environment (Rast et al., 2016). The VMB of healthy women (composed mainly of lactobacilli) helps to physically protect vaginal mucosa against infections by sustaining a low pH, producing lactic acid, and producing other antimicrobial compounds (Petrova et al., 2015). VVC, mainly caused by *C. albicans*, is an extremely common multifactorial mucosal infection that affects the lower female reproductive system and causes pathologic inflammation (Willems et al., 2020). In this study, we verified the ability of *C. albicans* to induce damage in vaginal epithelial cells in a rat model through histological and morphometric studies.

The fact that any epithelial cell layer that comes into contact with *C. albicans in vitro* is quickly and effectively invaded and destroyed by means of cell necrosis in the absence of a microbiota demonstrates the crucial role of the microbiome in opposing epithelial cell damage induced by *C. albicans* infection (Allert et al., 2018). Numerous studies have revealed that providing *lactobacillus* strains with beneficial characteristics alone or as a supplement to the standard antifungal therapies can be a successful method for preventing or treating VVC (Reid et al., 2006; Ehrström et al., 2010; Vladareanu et al., 2018). Various healthy *Lactobacillus* strains can exhibit a range of characteristics and effects on *Candida* species (Rodríguez-Arias et al., 2022). As a result, *in vitro* testing and animal experiments are essential for choosing the most promising *Lactobacillus* species with candidacidal effects (Rönnqvist et al., 2007; Joo et al., 2012; Wang et al., 2017). Previous *in vitro* studies demonstrated that the anticandidal effect of *L. acidophilus* could be attributed to different mechanisms including the inhibition of *C. albicans* biofilm formation and filamentation (Vilela et al., 2015; Matsubara et al., 2016), induction of coaggregation (Gil et al., 2010; Salari and Ghasemi Nejad Almani, 2020), and production of lactic acid (Gil et al., 2010; Matsubara et al., 2016) and hydrogen peroxide (Gil et al., 2010).

Different authors have investigated the *in vitro* anticandidal properties displayed by *L. johnsonii*. Vazquez-Munoz et al. reported that *L. johnsonii* reduced the growth of *C. albicans* and its capacity to change into hyphae and create biofilms (Vazquez-Munoz et al., 2022). Moreover, Charlet et al. found that *in vitro* co-incubation of *Candida* with *L. johnsonii* reduced the viability and proliferation of *C. albicans* by producing enzymes with chitinase-like action which facilitate the breakdown of chitin and the eradication of *C. albicans* (Charlet et al., 2020). Furthermore, Gil et al. reported that *L. johnsonii* displayed traits that made it a promising candidate for a probiotic strain, including its ability to co-aggregate with *Candida* species, attach to epithelial mucosa, and release both lactic acid and H_2_O_2_ (Gil et al., 2010). Through studying animal experimental models of VVC, the significance of inflammatory and immunological reactions necessary for the effective management of human infection and the factors that determine fungal pathogenicity have been highlighted (Cassone and Sobel, 2016). In this study, we selected *L*. *johnsinii* (B-2178) to conduct *in vivo* assays to compare its antifungal effect with the previously investigated *L. acidophilus* (LA-5^®^) in a rat model with VVC. In this work, we demonstrated that intravaginal delivery of *L. johnsonii* (B-2178) has a similar effect to *L. acidophilus* (LA-5^®^) in reducing vaginal *Candida* colonization as they both markedly decreased the *C. albicans* count in the lactobacilli-treated groups with a statistically significant difference compared to the untreated group even after 7 days of stoppage of treatment. Consequently, this animal model offers convincing proof of the candidacidal effects of *L. johnsonii* (B-2178) which was similar to the previously investigated *L. acidophilus* (LA-5^®^). Our findings are consistent with previous *in vivo* studies that evaluated the anticandidal effects of *L. acidophilus* as well as human clinical trials which confirmed the potential role of this probiotic in VVC. According to Vilela et al., the therapeutic or prophylactic use of *L. acidophilus* decreased the number of yeast cells in the *Galleria mellonella* larval hemolymph that was infected with *C. albicans* and enhanced the lifespan of these animals (Vilela et al., 2015). Similar results have been reported by Matsubara et al. who reported that the treatment of immunocompromised mice with probiotic *L. acidophilus* significantly minimized oral colonization with *C. albicans* compared to untreated mice (Matsubara et al., 2012).

In addition, many human clinical trials reported that different strains of *L. acidophilus* when administrated in a simple combination, could increase therapy efficacy, and reduce recurrences among VVC patients (Murina et al., 2014; Kovachev and Vatcheva-Dobrevska, 2015; Russo et al., 2019). In human clinical trials, *L. johnsonii* La1 has been used as a single agent probiotic, with various effective claims to reduce *H. pylori* carriage and to control allergic diseases. As far as we know, this is the initial work to assess the *in vivo* effect of *L. johnsonii* in a VVC animal model which will pave the path to its use in human clinical trials.

Numerous virulence features expressed by *C. albicans* contribute to the pathogenesis of VVC. Through two main mechanisms, this opportunistic pathogen can adhere to, infiltrate, and destroy cells in the vaginal mucosa: the secretion of virulence factors like Als family and Ssa1p which mediate biofilm formation and induce endocytosis of *Candida* by epithelial cells (Rast et al., 2016; Gao et al., 2019; Cangui-Panchi et al., 2023) and direct invasion by hyphal filaments, which are essential for penetrating mucosal defenses and damaging tissue by the release of degrading enzymes released at the hyphal tip and pressure imposed by the elongating filament (Lew, 2011; Sudbery, 2011). In our study, there was evident hyphae formation in the infected untreated group, while fewer hyphae formations were observed in both the lactobacilli-treated groups compared to the infected untreated group. However, hyphae formation was significantly reduced in the *L. johnsonii* (B-2178) treated group compared to the *L. acidophilus* (LA-5^®^) group which confirms their role in inhibiting hyphae formation in *C. albicans.*

VVC susceptibility was long thought to be caused by deficiencies in the adaptive immune response like those in other mucosal candidiasis types where susceptibility was found to be T cell-dependent. However, several clinical investigations and evidence from animal models showed that humoral or cell-mediated immune responses did not play any clear protective functions (Fidel, 2002; Samaranayake et al., 2002). In a previous investigation utilizing human volunteers, the recruitment of polymorphonuclear leukocytes into the vaginal lumen was found to be positively linked with reported disease manifestations, demonstrating that VVC was mediated through innate immune responses (Fidel et al., 2004). Considering the detrimental effect of host immunity on the development of the disease, VVC was classified as an immunopathology (Willems et al., 2020).

In an earlier study, Hickey et al. found that cells of the immune system, such as neutrophils, macrophages, and natural killer (NK) cells, are activated and multiplied within vaginal epithelial cells throughout the infective process, in addition to the development of micro-abscess (Hickey et al., 2011), which was observed in Group 3 in the current study. In our study, *L. acidophilus* (LA-5^®^) treatment resulted in minimal improvement, while treatment with *L. johnsonii* (B-2178) restored normal vaginal architecture apart from minimal neutrophil infiltration.

NF-κB is a fundamental transcription factor that controls the genes involved in both the innate and the adaptive immunological response. Moreover, the NF-κB signaling pathway is responsible for the expression of proinflammatory cytokines (Su et al., 2021). It is also crucial for regulating the survival, activation, and differentiation of inflammatory T lymphocytes. It triggers T-helper 17 (Th17) cells to release IL-17, a multifunctional pro-inflammatory cytokine, which promotes an effective inflammatory response through enhancing neutrophil recruitment and expression of cytokines (Conti et al., 2016). Furthermore, through regulating T cell receptor signaling, NF-κB induces Th1 cell differentiation which secrets IFN-γ, a cytokine that both enhances cellular immunity and participates in inflammatory processes (Oh and Ghosh, 2013). In the present study, *Candida* infection was associated with a significant increase in the optical density of NF-κB immunostaining of vaginal tissue in the infected untreated animal group compared to the uninfected groups. This could be explained by Oky et al. who reported that epithelial cells activate NF-κB in response to *Candida* colonization via a Toll-like receptor-4 (TLR-4) dependent pathway (Oky et al., 2020). Previous research has shown that suppressing NF-κB signaling is a major mechanism contributing to the anti-inflammatory function of lactobacilli (Rottenberg et al., 2002) Another investigation supported the idea that lactobacilli could prevent TLR-4-associated NF-κB activation (Lee et al., 2009). In our work, the optical density of NF-κB immunostaining of vaginal tissue was significantly reduced in both the lactobacilli-treated groups compared to the infected untreated group. However, the inhibitory effect of *L. johnsonii* (B-2178) on NF-κB was significantly higher compared to *L. acidophilus* (LA-5^®^). Our data are consistent with Santos et al. who observed that *C. albicans* activated NF-κB in HeLa cells, but *L. plantarum* and *L. fermentum* therapy inhibited its activation (Santos et al., 2018). Additionally, *L. rhamnosus and L. reuteri* inhibited NF-κB signaling by suppressing *C. albicans-*induced NF-κB IκB kinase (Ikka) in VK2/E667 cells (Wagner and Johnson, 2012).

Cytokines create a hostile environment for the pathogen by interacting with many immune cells or by inducing an antimicrobial response (Hickey et al., 2011). In the current study, the inflammatory cytokines IFN-γ (the hallmark of Th1) as well as IL-17 were significantly higher in the vaginal tissue of infected untreated animals compared to the control uninfected groups, while IL-4 (the hallmark of Th-2) was decreased in the vaginal tissue of these animals. This shows that our infection model has induced a dominating Th1 cytokine response and is consistent with the detected pattern of the NF-κB signaling pathway in the vaginal tissue. Our results are in line with results reported in a previous rat model study, where no IL-4 or IL-5 was found in the vaginal fluid after inoculation, while a notable Th1 cytokine pattern was found during the primary infection (de Bernardis et al., 2000).

Most of the negative health effects linked to VVC are caused by a short-term or long-term inflammatory response (Fidel et al., 2004). The capability of controlling inflammation is essential for maintaining a balance between immunopathology and protection in mucosal infections. Therefore, for a *Lactobacillus* strain to be considered as a possible probiotic candidate, it is essential to ascertain its anti-inflammatory effects (Petrova et al., 2015). In the current study, both *L. johnsonii* (B-2178) and *L. acidophilus* (LA-5^®^) redirected the immunological profile to a tolerant or regulating state that maintains a balanced Th1/Th2 ratio and inhibits the generation of proinflammatory cytokines by changing the cytokine profiles of vaginal epithelial cells. In numerous *in vitro* studies, a significant downregulation of Th1 cytokines; IL-2, IFN-γ and the proinflammatory IL-17 by probiotic lactobacilli strains has been reported (Schultz et al., 2002; Lee et al., 2009; Llopis et al., 2009; Bäuerl et al., 2013). Moreover, a recent *in vivo* study in murine models confirmed that lactobacilli have a moderating influence on cytokine production during *Candida* infection where intravaginal administration of *L. crispatus* and *L. delbrueckii* lowered IFN-γ and IL-17 while increased IL-4 expression in vaginal tissue (Li et al., 2019). Clinical studies demonstrate that lactobacilli have immune-modulating effects in humans and that giving probiotics to pregnant women with VVC reduced the duration of inflammation over time compared to the control group (Ang et al., 2022).

According to Nisaa et al., transforming growth factor (TGF)- α is a critical mediator of acute inflammation and is considerably elevated in *Candida* vaginal infection (Nisaa et al., 2023). TGF-α is involved in several processes during wound healing, including inflammation, activating angiogenesis, fibroblast proliferation and collagen production, and deposition (Nall et al., 1996). This explains the increase in the mean area percentage of collagen fibers stained with the trichrome stain after *Candida* infection in our study. However, in the present study, the vaginas of infected untreated animals displayed destructed elastic fibers, leaving a few fine fibers, which could be explained by Oky et al. who stated that following *C. albicans* infection, IL-6 cytokine levels increased in rats, throughout the inflammatory phase, which led to the breakdown of elastic fibers (Oky et al., 2020). Visser et al. (2022) reported that there is a substantial link between IL-6 and the degeneration of elastic fibers. In our study, it was reported that both the lactobacilli-treated groups exhibited a significant decrease in the mean area percentage of collagen fibers compared to the untreated group. However, the CT stroma of the *L. johnsonii* (B-2178) treated group demonstrated long branching elastic fibers with a significantly reduced number of collagen fibers compared to the *L. acidophilus* (LA-5^®^) treated group.

A recent study conducted by Pacha-Herrera et al. (2022) reported a strong multi-microbial interaction between different lactobacilli species. Their study demonstrated that the absence of *L. acidophilus* in other lactobacilli clusters can lead to defective probiotic protection in vaginal dysbiosis. Therefore, although in our study *L. johnsonii* was superior to *L. acidophilus* regarding its effect in reducing both vaginal epithelial damage and hyphae formation, the protective probiotic role of *L. acidophilus* as a part of a probiotic multi-microbial consortium should not be overlooked.

## 5. Conclusion

Our study verified that *L. johnsonii* (B-2178) has a similar effect to *L. acidophilus* (LA-5^®^) in the reduction of *C. albicans* vaginal colonization as well as in reducing the pro-inflammatory cytokines IL-17 and IFN-γ (the hallmark of Th1), suggesting an immune tolerant state which is maintained in the vaginal epithelium by *lactobacillus* colonization. However, treatment with *L. johnsonii* (B-2178) significantly minimized the epithelium damage triggered by *C. albicans* infection and restored normal vaginal architecture as evidenced by the histologic and morphometric studies compared to *L. acidophilus* (LA-5^®^), partly due to significant reduction of hyphae formation and partly due to ameliorating the undesirable uncontrolled inflammatory response in the vaginal tissue. Our study has some limitations. First, we did not assess the combined effect of *L. johnsonii* and *L. acidophilus* against *C. albicans* vs. the effect of each individual strain. Second, we did not evaluate the effect of lactobacilli species on the treatment or prevention of recurrences of VVC. Therefore, further studies are required to assess the combined effect of both lactobacilli species compared to their individual activity, to investigate the precise mechanism of the anticandidal effect of the lactobacilli strains used, and to explore their ability to treat recurrent VVC in animal models. Despite limitations, the promising results of our study highlighted the potential of *L. johnsonii* (B-2178) to be used either alone or in combination with other lactobacilli species in human clinical trials, utilizing their observed characteristics to treat or prevent VVC including recurrent forms.

## Data availability statement

The original contributions presented in the study are included in the article/Supplementary material, further inquiries can be directed to the corresponding author.

## Ethics statement

The animal study was reviewed and approved by Research Ethics Committee at October 6 University (NO. PRE-Me-2212045).

## Author contributions

DE, AA, and AS: conceptualization. DE, AA, AS, and SH: methodology and validation. DE, HM, AS, and SH: writing—original draft and figure preparation. DE, AA, HM, AS, and SH: writing—review and editing. DE and AA: supervision. All authors contributed to the article and approved the submitted version.

## Conflict of interest

The authors declare that the research was conducted in the absence of any commercial or financial relationships that could be construed as a potential conflict of interest.

## Publisher’s note

All claims expressed in this article are solely those of the authors and do not necessarily represent those of their affiliated organizations, or those of the publisher, the editors and the reviewers. Any product that may be evaluated in this article, or claim that may be made by its manufacturer, is not guaranteed or endorsed by the publisher.

## References

[ref1] Abd El-Aala A. A. Ismail M. A. Awad A. R. El Sharkawy M. A. (2023). Effect of Lactobacillus acidophilus and *Lactobacillus johnsonii* on growth, phenotypic and genotypic expression of virulence factors of *Candida albicans* causing vulvovaginal candidiasis. Egypt. J. Med. Microbiol. 32, 41–49. doi: 10.21608/ejmm.2023.299696

[ref2] Achkar J. M. Fries B. C. (2010). Candida infections of the genitourinary tract. Clin. Microbiol. Rev. 23, 253–273. doi: 10.1128/CMR.00076-09, PMID: 20375352 PMC2863365

[ref3] Allert S. Förster T. M. Svensson C.-M. Richardson J. P. Pawlik T. Hebecker B.. (2018). *Candida albicans*-induced epithelial damage mediates translocation through intestinal barriers. MBio 9:e00915-18. doi: 10.1128/mBio.00915-18, PMID: 29871918 PMC5989070

[ref4] Ang X. Y. Mageswaran U. M. Chung Y. L. F. Lee B. K. Azhar S. N. A. Roslan N. S.. (2022). Probiotics reduce vaginal candidiasis in pregnant women via modulating abundance of Candida and Lactobacillus in vaginal and Cervicovaginal regions. Microorganisms 10:285. doi: 10.3390/microorganisms10020285, PMID: 35208739 PMC8877909

[ref5] Assefa S. Ahles K. Bigelow S. Curtis J. T. Köhler G. A. (2015). Lactobacilli with probiotic potential in the prairie vole (*Microtus ochrogaster*). Gut Pathog. 7:35. doi: 10.1186/s13099-015-0082-0, PMID: 26719773 PMC4696317

[ref6] Bäuerl C. Llopis M. Antolín M. Monedero V. Mata M. Zúñiga M.. (2013). Lactobacillus paracasei and *Lactobacillus plantarum* strains downregulate proinflammatory genes in an *ex vivo* system of cultured human colonic mucosa. Genes Nutr. 8, 165–180. doi: 10.1007/s12263-012-0301-y, PMID: 22669626 PMC3575885

[ref7] Begum N. Manipriya K. Begum R. (2020). Simple and rapid method for rat estrous cycle identification using crystal violet-hormonal consideration. Int. J. Appl. Pharm. Sci. Res. 5, 54–59. doi: 10.21477/ijapsr.5.4.1,B, V

[ref8] Cangui-Panchi S. P. Ñacato-Toapanta A. L. Enríquez-Martínez L. J. Salinas-Delgado G. A. Reyes J. Garzon-Chavez D.. (2023). Battle royale: immune response on biofilms – host-pathogen interactions. Curr. Res. Immunol. 4:100057. doi: 10.1016/j.crimmu.2023.100057, PMID: 37025390 PMC10070391

[ref9] Carrara M. A. Donatti L. Damke E. Svidizinski T. I. E. Consolaro M. E. L. Batista M. R. (2010). A new model of vaginal infection by *Candida albicans* in rats. Mycopathologia 170, 331–338. doi: 10.1007/s11046-010-9326-1 20532984

[ref10] Cassone A. Sobel J. D. (2016). Experimental models of vaginal candidiasis and their relevance to human candidiasis. Infect. Immun. 84, 1255–1261. doi: 10.1128/IAI.01544-15, PMID: 26883592 PMC4862719

[ref11] Chan Y. H. (2003). Biostatistics 102: quantitative data-parametric & non-parametric tests. Singapore Med. J. 44, 391–396. 14700417

[ref12] Chan Y. H. (2004). Biostatistics 301. Repeated measurement analysis. Singapore Med. J. 45, 354–368. 15284929

[ref13] Charlet R. Bortolus C. Sendid B. Jawhara S. (2020). Bacteroides thetaiotaomicron and *Lactobacillus johnsonii* modulate intestinal inflammation and eliminate fungi via enzymatic hydrolysis of the fungal cell wall. Sci. Rep. 10:11510. doi: 10.1038/s41598-020-68214-9, PMID: 32661259 PMC7359362

[ref14] Chee W. J. Y. Chew S. Y. Than L. T. L. (2020). Vaginal microbiota and the potential of Lactobacillus derivatives in maintaining vaginal health. Microb. Cell Factories 19:203. doi: 10.1186/s12934-020-01464-4, PMID: 33160356 PMC7648308

[ref15] Conti H. R. Bruno V. M. Childs E. E. Daugherty S. Hunter J. P. Mengesha B. G.. (2016). IL-17 receptor signaling in Oral epithelial cells is critical for protection against oropharyngeal candidiasis. Cell Host Microbe 20, 606–617. doi: 10.1016/j.chom.2016.10.001 27923704 PMC5147498

[ref16] de Bernardis F. Santoni G. Boccanera M. Spreghini E. Adriani D. Morelli L.. (2000). Local Anticandidal immune responses in a rat model of vaginal infection by and protection against *Candida albicans*. Infect. Immun. 68, 3297–3304. doi: 10.1128/IAI.68.6.3297-3304.2000, PMID: 10816477 PMC97585

[ref17] Denning D. W. Kneale M. Sobel J. D. Rautemaa-Richardson R. (2018). Global burden of recurrent vulvovaginal candidiasis: a systematic review. Lancet Infect. Dis. 18, e339–e347. doi: 10.1016/S1473-3099(18)30103-8, PMID: 30078662

[ref18] Ehrström S. Daroczy K. Rylander E. Samuelsson C. Johannesson U. Anzén B.. (2010). Lactic acid bacteria colonization and clinical outcome after probiotic supplementation in conventionally treated bacterial vaginosis and vulvovaginal candidiasis. Microbes Infect. 12, 691–699. doi: 10.1016/j.micinf.2010.04.010, PMID: 20472091

[ref21] FAO/WHO, (2006). Probiotics in food: health and nutritional properties and guidelines for evaluation. fao food and nutrition paper 85, food and agriculture organization of the united nations, Rome: World Health Organization.

[ref19] Fidel P. L. J. (2002). Distinct protective host defenses against oral and vaginal candidiasis. Med. Mycol. 40, 359–375. doi: 10.1080/714031126, PMID: 12230215

[ref20] Fidel P. L. Barousse M. Espinosa T. Ficarra M. Sturtevant J. Martin D. H.. (2004). An intravaginal live Candida challenge in humans leads to new hypotheses for the Immunopathogenesis of vulvovaginal candidiasis. Infect. Immun. 72, 2939–2946. doi: 10.1128/IAI.72.5.2939-2946.2004, PMID: 15102806 PMC387876

[ref22] Fujisawa T. Benno Y. Yaeshima T. Mitsuoka T., and Mitsuoka, T. (1992). Taxonomic study of the *Lactobacillus acidophilus* group, with recognition of *Lactobacillus gallinarum* sp. nov. and *Lactobacillus johnsonii* sp. nov. and synonymy of *Lactobacillus acidophilus* group A3 (Johnson et al. 1980) with the type strain of Lactobacill. Int. J. Syst. Bacteriol. 42, 487–491. doi:10.1099/00207713-42-3-487, PMID: 1503977

[ref23] Gao Y. Liang G. Wang Q. She X. Shi D. Shen Y.. (2019). Different host immunological response to *C. albicans* by human Oral and vaginal epithelial cells. Mycopathologia 184, 1–12. doi: 10.1007/s11046-018-0301-6, PMID: 30600418

[ref24] Gil N. F. Martinez R. C. R. Gomes B. C. Nomizo A. De Martinis E. C. P. (2010). Vaginal lactobacilli as potential probiotics against Candida spp. Brazilian J. Microbiol. 41, 6–14. doi: 10.1590/S1517-83822010000100002, PMID: 24031455 PMC3768620

[ref25] Hickey D. K. Patel M. V. Fahey J. V. Wira C. R. (2011). Innate and adaptive immunity at mucosal surfaces of the female reproductive tract: stratification and integration of immune protection against the transmission of sexually transmitted infections. J. Reprod. Immunol. 88, 185–194. doi: 10.1016/j.jri.2011.01.005, PMID: 21353708 PMC3094911

[ref26] Hickey R. J. Zhou X. Pierson J. D. Ravel J. Forney L. J. (2012). Understanding vaginal microbiome complexity from an ecological perspective. Transl. Res. 160, 267–282. doi: 10.1016/j.trsl.2012.02.008, PMID: 22683415 PMC3444549

[ref27] Jang S. J. Lee K. Kwon B. You H. J. Ko G. (2019). Vaginal lactobacilli inhibit growth and hyphae formation of *Candida albicans*. Sci. Rep. 9:8121. doi: 10.1038/s41598-019-44579-4, PMID: 31148560 PMC6544633

[ref28] Joo H.-M. Hyun Y.-J. Myoung K.-S. Ahn Y.-T. Lee J.-H. Huh C.-S.. (2011). *Lactobacillus johnsonii* HY7042 ameliorates *Gardnerella vaginalis*-induced vaginosis by killing Gardnerella vaginalis and inhibiting NF-κB activation. Int. Immunopharmacol. 11, 1758–1765. doi: 10.1016/j.intimp.2011.07.002, PMID: 21798373

[ref29] Joo H.-M. Kim K.-A. Myoung K.-S. Ahn Y.-T. Lee J.-H. Huh C.-S.. (2012). *Lactobacillus helveticus* HY7801 ameliorates vulvovaginal candidiasis in mice by inhibiting fungal growth and NF-κB activation. Int. Immunopharmacol. 14, 39–46. doi: 10.1016/j.intimp.2012.05.023, PMID: 22735758

[ref30] Kovachev S. M. Vatcheva-Dobrevska R. S. (2015). Local probiotic therapy for vaginal *Candida albicans* infections. Probiotics Antimicrob. Proteins 7, 38–44. doi: 10.1007/s12602-014-9176-0, PMID: 25362524

[ref31] Lee H. Ahn Y.-T. Lee J.-H. Huh C.-S. Kim D.-H. (2009). Evaluation of anti-colitic effect of lactic acid Bacteria in mice by cDNA microarray analysis. Inflammation 32, 379–386. doi: 10.1007/s10753-009-9146-y, PMID: 19711178

[ref32] Lew R. R. (2011). How does a hypha grow? The biophysics of pressurized growth in fungi. Nat. Rev. Microbiol. 9, 509–518. doi: 10.1038/nrmicro2591, PMID: 21643041

[ref33] Li T. Liu Z. Zhang X. Chen X. Wang S. (2019). Local probiotic lactobacillus crispatus and *lactobacillus delbrueckii* exhibit strong antifungal effects against vulvovaginal candidiasis in a rat model. Front. Microbiol. 10:1033. doi: 10.3389/fmicb.2019.01033, PMID: 31139166 PMC6519388

[ref34] Llopis M. Antolin M. Carol M. Borruel N. Casellas F. Martinez C.. (2009). *Lactobacillus casei* downregulates commensalsʼ inflammatory signals in Crohnʼs disease mucosa. Inflamm. Bowel Dis. 15, 275–283. doi: 10.1002/ibd.20736, PMID: 18839424

[ref35] Mandl A. M. (1951). The phases of the Oestrous cycle in the adult white rat. J. Exp. Biol. 28, 576–584. doi: 10.1242/jeb.28.4.576

[ref36] Marcial G. E. Ford A. L. Haller M. J. Gezan S. A. Harrison N. A. Cai D.. (2017). *Lactobacillus johnsonii* N6.2 modulates the host immune responses: A double-blind, randomized trial in healthy adults. Front. Immunol. 8:655. doi: 10.3389/fimmu.2017.00655, PMID: 28659913 PMC5466969

[ref37] Matsubara V. Silva E. Paula C. Ishikawa K. Nakamae A. (2012). Treatment with probiotics in experimental oral colonization by *Candida albicans* in murine model (DBA/2). Oral Dis. 18, 260–264. doi: 10.1111/j.1601-0825.2011.01868.x, PMID: 22059932

[ref38] Matsubara V. H. Wang Y. Bandara H. M. H. N. Mayer M. P. A. Samaranayake L. P. (2016). Probiotic lactobacilli inhibit early stages of *Candida albicans* biofilm development by reducing their growth, cell adhesion, and filamentation. Appl. Microbiol. Biotechnol. 100, 6415–6426. doi: 10.1007/s00253-016-7527-3, PMID: 27087525

[ref39] Murina F. Graziottin A. Vicariotto F. De Seta F. (2014). Can *Lactobacillus fermentum* LF10 and *Lactobacillus acidophilus* LA02 in a slow-release vaginal product be useful for prevention of recurrent vulvovaginal candidiasis? A clinical study. Available at: www.jcge.com. 10.1097/MCG.0000000000000225 25291115

[ref40] Nall A. V. Brownlee R. E. Colvin C. P. Schultz G. Fein D. Cassisi N. J.. (1996). Transforming growth factor 1 improves wound healing and random flap survival in Normal and irradiated rats. Arch. Otolaryngol. Head Neck Surg. 122, 171–177. doi: 10.1001/archotol.1996.01890140057011, PMID: 8630211

[ref41] Nisaa A. A. Oon C.-E. Sreenivasan S. Balakrishnan V. Tan J. J. Teh C. S.-J.. (2023). Breast milk from healthy women has higher anti-Candida properties than women with vaginal infections during pregnancy. Food Sci. Biotechnol. 32, 471–480. doi: 10.1007/s10068-022-01088-x 36911325 PMC9992674

[ref42] Oh H. Ghosh S. (2013). NF-κB: roles and regulation in different CD4 ^+^ T-cell subsets. Immunol. Rev. 252, 41–51. doi: 10.1111/imr.12033, PMID: 23405894 PMC3576882

[ref43] Oky P. Tania A. Biomedik B. Biomolekuler P. Kedokteran F. Wijaya U.. (2020). Mekanisme Escape dan Respon Imun innate terhadap *Candida albicans*. Jurnal Ilmiah Kedokteran Wijaya Kusuma 9:60. doi: 10.30742/jikw.v9i1.747

[ref44] Pacha-Herrera D. Erazo-Garcia M. P. Cueva D. F. Orellana M. Borja-Serrano P. Arboleda C.. (2022). Clustering analysis of the multi-microbial consortium by Lactobacillus species against vaginal Dysbiosis among Ecuadorian women. Front. Cell. Infect. Microbiol. 12, 1–10. doi: 10.3389/fcimb.2022.863208, PMID: 35646732 PMC9131875

[ref45] Petrova M. I. Lievens E. Malik S. Imholz N. Lebeer S. (2015). Lactobacillus species as biomarkers and agents that can promote various aspects of vaginal health. Front. Physiol. 6:81. doi: 10.3389/fphys.2015.00081, PMID: 25859220 PMC4373506

[ref46] Rast T. J. Kullas A. L. Southern P. J. Davis D. A. (2016). Human epithelial cells discriminate between commensal and pathogenic interactions with *Candida albicans*. PLoS One 11:e0153165. doi: 10.1371/journal.pone.0153165, PMID: 27088599 PMC4835109

[ref47] Reid G. Kim S. O. Köhler G. A. (2006). Selecting, testing and understanding probiotic microorganisms. FEMS Immunol. Med. Microbiol. 46, 149–157. doi: 10.1111/j.1574-695X.2005.00026.x 16487295

[ref48] Rodríguez-Arias R. J. Guachi-Álvarez B. O. Montalvo-Vivero D. E. Machado A. (2022). Lactobacilli displacement and *Candida albicans* inhibition on initial adhesion assays: a probiotic analysis. BMC. Res. Notes 15:239. doi: 10.1186/s13104-022-06114-z, PMID: 35799214 PMC9264498

[ref49] Rönnqvist D. Forsgren-Brusk U. Husmark U. Grahn-Håkansson E. (2007). *Lactobacillus fermentum* Ess-1 with unique growth inhibition of vulvo-vaginal candidiasis pathogens. J. Med. Microbiol. 56, 1500–1504. doi: 10.1099/jmm.0.47226-0, PMID: 17965352

[ref50] Rottenberg S. Schmuckli-Maurer J. Grimm S. Heussler V. T. Dobbelaere D. A. (2002). Characterization of the bovine IκB kinases (IKK)α and IKKβ, the regulatory subunit NEMO and their substrate IκBα. Gene 299, 293–300. doi: 10.1016/S0378-1119(02)01011-9, PMID: 12459277

[ref51] Russo R. Superti F. Karadja E. De Seta F. (2019). Randomised clinical trial in women with recurrent vulvovaginal candidiasis: efficacy of probiotics and lactoferrin as maintenance treatment. Mycoses 62, 328–335. doi: 10.1111/myc.12883, PMID: 30565745

[ref52] Salari S. Ghasemi Nejad Almani P. (2020). Antifungal effects of Lactobacillus acidophilus and *Lactobacillus plantarum* against different oral Candida species isolated from HIV/AIDS patients: an *in vitro* study. J. Oral Microbiol. 12:1769386. doi: 10.1080/20002297.2020.1769386, PMID: 32922676 PMC7448839

[ref53] Samaranayake L. Fidel P. Naglik Sweet S. Teanpaisan R. Coogan M. Blignaut E.. (2002). Fungal infections associated with HIV infection. Oral Dis. 8, 151–160. doi: 10.1034/j.1601-0825.8.s2.6.x 12164650

[ref54] Santos C. M. A. Pires M. C. V. Leão T. L. Silva A. K. S. Miranda L. S. Martins F. S.. (2018). Anti-inflammatory effect of two Lactobacillus strains during infection with gardnerella vaginalis and *Candida albicans* in a hela cell culture model. Microbiology (United Kingdom) 164, 349–358. doi: 10.1099/mic.0.000608 29458690

[ref55] Schultz M. Veltkamp C. Dieleman L. A. Grenther W. B. Wyrick P. B. Tonkonogy S. L.. (2002). *Lactobacillus plantarum* 299V in the treatment and prevention of spontaneous colitis in Interleukin-10-deficient mice. Inflamm. Bowel Dis. 8, 71–80. doi: 10.1097/00054725-200203000-00001, PMID: 11854603

[ref56] Souza V. R. Mendes E. Casaro M. Antiorio A. T. F. B. Oliveira F. A. Ferreira C. M. (2019). Description of ovariectomy protocol in mice. Methods Mol. Biol., 303–309. doi: 10.1007/978-1-4939-8994-2_29 30535707

[ref57] Spaggiari L. Sala A. Ardizzoni A. De Seta F. Singh D. K. Gacser A.. (2022). *Lactobacillus acidophilus*, *L. plantarum*, L. rhamnosus, and *L. reuteri* cell-free supernatants inhibit *Candida parapsilosis* pathogenic potential upon infection of vaginal epithelial cells monolayer and in a Transwell Coculture system *in vitro*. Microbiol. Spectr. 10, 10:e0269621. doi: 10.1128/spectrum.02696-21, PMID: 35499353 PMC9241606

[ref58] Stabile G. Gentile R. M. Carlucci S. Restaino S. De Seta F. (2021). A new therapy for uncomplicated vulvovaginal candidiasis and its impact on vaginal flora. Healthcare 9:1555. doi: 10.3390/healthcare9111555, PMID: 34828601 PMC8625853

[ref59] Strus M. Kucharska A. Kukla G. Brzychczy-Włoch M. Maresz K. Heczko P. B. (2005). The *in vitro* activity of vaginal Lactobacillus with probiotic properties against Candida. Infect. Dis. Obstet. Gynecol. 13, 69–75. doi: 10.1080/10647440400028136, PMID: 16011996 PMC1784560

[ref60] Su C.-M. Wang L. Yoo D. (2021). Activation of NF-κB and induction of proinflammatory cytokine expressions mediated by ORF7a protein of SARS-CoV-2. Sci. Rep. 11:13464. doi: 10.1038/s41598-021-92941-2, PMID: 34188167 PMC8242070

[ref61] Sudbery P. E. (2011). Growth of *Candida albicans* hyphae. Nat. Rev. Microbiol. 9, 737–748. doi: 10.1038/nrmicro2636 21844880

[ref62] Vazquez-Munoz R. Dongari-Bagtzoglou A. (2021). Anticandidal activities by Lactobacillus species: an update on mechanisms of action. Front. Oral Heal. 2:689382. doi: 10.3389/froh.2021.689382, PMID: 35048033 PMC8757823

[ref63] Vazquez-Munoz R. Thompson A. Russell J. T. Sobue T. Zhou Y. Dongari-Bagtzoglou A. (2022). Insights from the *Lactobacillus johnsonii* genome suggest the production of metabolites with Antibiofilm activity against the Pathobiont *Candida albicans*. Front. Microbiol. 13:853762. doi: 10.3389/fmicb.2022.853762, PMID: 35330775 PMC8940163

[ref64] Vilela S. F. Barbosa J. O. Rossoni R. D. Santos J. D. Prata M. C. Anbinder A. L.. (2015). *Lactobacillus acidophilus* ATCC 4356 inhibits biofilm formation by C. albicans and attenuates the experimental candidiasis in galleria mellonella. Virulence 6, 29–39. doi: 10.4161/21505594.2014.981486, PMID: 25654408 PMC4603435

[ref65] Visser M. P. J. Dofferhoff A. S. M. van den Ouweland J. M. W. van Daal H. Kramers C. Schurgers L. J.. (2022). Effects of vitamin D and K on Interleukin-6 in COVID-19. Front. Nutr. 8:761191. doi: 10.3389/fnut.2021.761191 35111793 PMC8801698

[ref66] Vladareanu R. Mihu D. Mitran M. Mehedintu C. Boiangiu A. Manolache M.. (2018). New evidence on oral *L. plantarum* P17630 product in women with history of recurrent vulvovaginal candidiasis (RVVC): a randomized double-blind placebo-controlled study. Eur. Rev. Med. Pharmacol. Sci. 22, 262–267. doi: 10.26355/eurrev_201801_14128, PMID: 29364495

[ref67] Wagner R. Johnson S. J. (2012). Probiotic lactobacillus and estrogen effects on vaginal epithelial gene expression responses to *Candida albicans*. J. Biomed. Sci. 19:58. doi: 10.1186/1423-0127-19-58, PMID: 22715972 PMC3404894

[ref68] Wang S. Wang Q. Yang E. Yan L. Li T. Zhuang H. (2017). Antimicrobial compounds produced by vaginal *Lactobacillus crispatus* are able to strongly inhibit *Candida albicans* growth, hyphal formation and regulate virulence-related gene expressions. Front. Microbiol. 8:564. doi: 10.3389/fmicb.2017.00564, PMID: 28421058 PMC5378977

[ref69] Willems H. M. E. Ahmed S. S. Liu J. Xu Z. Peters B. M. (2020). Vulvovaginal candidiasis: A current understanding and burning questions. J. Fungi 6:27. doi: 10.3390/jof6010027, PMID: 32106438 PMC7151053

[ref70] XieHY F. F. (2017). Cochrane library Cochrane database of systematic reviews probiotics for vulvovaginal candidiasis in non-pregnant women (review). Cochrane Database Syst. Rev. 11:CD010496. doi: 10.1002/14651858.CD010496.pub2, PMID: 29168557 PMC6486023

[ref71] Yefet E. Colodner R. Battino S. Wattad M. Nachum Z. (2022). Oral probiotics for the secondary prevention of vulvovaginal infections in pregnant women-double-blind, randomized, placebo-controlled study. Am. J. Obstet. Gynecol. 226, S130–S131. doi: 10.1016/j.ajog.2021.11.231

[ref72] Zheng J. Wittouck S. Salvetti E. Franz C. M. A. P. Harris H. M. B. Mattarelli P.. (2020). A taxonomic note on the genus Lactobacillus: description of 23 novel genera, emended description of the genus Lactobacillus Beijerinck 1901, and union of Lactobacillaceae and Leuconostocaceae. Int. J. Syst. Evol. Microbiol. 70, 2782–2858. doi: 10.1099/ijsem.0.004107, PMID: 32293557

