# Supplementary material for: Comparing the therapeutic potentials of Lactobacillus johnsonii vs. Lactobacillus acidophilus against vulvovaginal candidiasis in female rats: an in vivo study
Source: Front Microbiol. 2023 Jul 17;14:1222503. doi: 10.3389/fmicb.2023.1222503 (PMC10388188; doi:10.3389/fmicb.2023.1222503)
Supplement: Supplementary file 1 [file Table_1.DOCX]

|  | **Group 1** | **Group 2** | **Group 3** | **Group 4** | **Group 5** |
| --- | --- | --- | --- | --- | --- |
| **Calculated count (before ttt)** | - | - | 4166.67±776.32 | 4966.67±852.45 | 4850±1419.51 |
| **Calculated count (1 day after stop ttt)** | - | - | 4333.33±1269.12 | 133.33±40.82 ¢, | 250±77.46 ¢, |
| **Calculated count (3 days after stop of ttt)** | - | - | 5000±1214.91 | 1150±137.84 ¢**,** | 1566.67±467.62 ¢**,** |
| **Calculated count (7 days after stop of ttt)** | - | - | 4750±1541.1 | 416.67±116.9 ¢^,^ | 400±126.49 ¢, |
| **IFN-γ (PG/gm)** | 102.92±9.34 | 255.77±13.98 * | 315.83±31.64 *# | 150.38±24.07 *#¢ | 147.65±24.13 *#¢ |
| **IL-17 (PG/gm)** | 381.55±39.62 | 547.72±58.12 * | 577.27±90.46 * | 434.57±28.29 #¢ | 439±37.37 #¢ |
| **IL-4 (PG/gm)** | 282.43±23.93 | 208.32±4.86 * | 145.88±35.17 *¢ | 227.87±40.73 *¢ | 229.17±28.34 *¢ |
| **Mean number of Hyphae** | 0±0 | 0±0 | 24.5±5.82 | 18.83±3.49 ¢ | 3.17±1.72 ¢ ß |
| **Mean area % of collagen fibers** | 2.32±0.29 | 2.49±0.24 | 13.54±0.39 | 10.9±0.4 ¢ | 2.23±0.29 ¢ ß |
| **Optical density of NF-kβ immunostaining** | 0.22±0.04 | 0.25±0.01 | 0.89±0.03 | 0.36±0.04 ¢ | 0.26±0.01 ¢ ß |
